# Safety of Conservative Management of High-Grade Squamous Intraepithelial Lesion in Women Under 30 Years Old

**DOI:** 10.1089/whr.2022.0024

**Published:** 2022-06-22

**Authors:** Mariana K. Bonas, Michelle G. Discacciati, Hisa M. Videira, Lucas A. Cavalcante, Julio C. Teixeira, Diama B. Vale

**Affiliations:** Obstetrics and Gynecology Department, University of Campinas, Campinas, Brazil.

**Keywords:** cervical intraepithelial neoplasia, papillomavirus infections, young adult, conservative treatment, uterine cervical neoplasms, secondary prevention

## Abstract

**Objectives:**

To evaluate the outcomes of conservative management in young women with high-grade squamous intraepithelial lesion (HSIL).

**Methods:**

A retrospective cohort study included women younger than 30 years referred with HSIL (cytology or biopsy) managed conservatively from 2012 to 2019, in Campinas/Brazil. Regression was the outcome when no evidence of HSIL was observed in at least two consecutive follow-ups. Kaplan–Meyer method was used to determine regression probabilities. Other tests were chi-square or Fisher, Mann–Whitney and COX regression.

**Results:**

During the study period, 89 patients were included. No progression to microinvasive or invasive cancer was observed. Sixty-one (69%) patients were younger than 25 years, and 28 (31%) were aged 25–30 years. Spontaneous regression was seen in 64 (72%) and persistence in 25 (28%) of the overall sample. The average time to regression was 15.4 months (standard deviation [SD] = 7.7), and the follow-up time was 31.6 months (SD 19.0). Age, parity, first sexual intercourse, smoking, hormonal contraception, and colposcopy impression were not different among women with regression or persistence. Regression probabilities were, respectively, 28.9%, 60.2%, and 78.1% after 12, 18, and 24 months. Most of the events happened between 12 and 18 months of follow-up.

**Conclusions:**

Conservative management in women younger than 30 years was safe: spontaneous regression was observed in 72% of all women younger than 30 with HSIL managed conservatively. No clinical variable was relevant, influencing regression. In 2 years the regression probability was 78%.

## Introduction

Cervical cancer is a global public health problem and affects thousands of women in their reproductive ages. The most common risk factor is persistent infection by oncogenic types of human papillomavirus (HPV), usually acquired after a sexual debut. Molecular mechanisms induced by the presence of the virus may result in genetic instability and clonal malignancy, leading to modifications in the cervical epithelium with the possibility of progression to invasive cancer.^1,2^ The precursor lesions are known as high-grade squamous intraepithelial lesions (HSIL) or cervical intraepithelial neoplasia grades 2–3 (CIN 2–3) and adenocarcinoma *in situ*. Their treatment is essential to prevent progression to cancer.^3^ The most common treatment recommended is excisional through large loop excision of the transformation zone (LLETZ).^4–6^ It is strongly recommended for adult women. However, for women younger than 30 years, there is a debate concerning the risks and benefits of excision.^5–7^

In young women, the squamous–columnar junction of the cervix is an area of constant metaplasia. At puberty, the alterations in the vaginal pH and hormone variations occur more intensely, offering favorable conditions for HPV infection.^8^ The majority of the infections in this group are self-limited.^9,10^ Furthermore, this group has a low rate of progression to invasive carcinoma.^10^ The self-limited profile of the HPV infection is sustained by high regression rates in women younger than 30 years, especially in those below 25 years.^11,12^

Although there is a clear benefit of LLETZ in preventing invasive cancer, there is a significant risk of unfavorable outcomes such as preterm birth, premature rupture of membranes, low birth weight, and neonatal mortality and morbidity, consistent with the deepness of the tissue removed.^7^ The risk of unfavorable pregnancy outcomes is more pronounced in younger patients, especially preterm labor.^13^

Many guidelines worldwide suggest that being conservative is a possible option for management.^4–6,14^ The rationale is based on the evidence that within the heterogeneous group lesions that compose HSIL, specific subgroups, particularly young women, behave like low-grade squamous intraepithelial lesions (LSIL).^10,15^ Smoking, multiparity, and prolonged use of oral contraceptives can affect the natural history of HSIL, increasing the risk of progression.^16^ This research aims to evaluate the outcomes of conservative management in young women at a reference center in Campinas, Brazil. We expect the results to support a safe recommendation considering the obstetric risks and overtreatment in young women.

## Materials and Methods

It is a retrospective cohort study that included all women under 30 years old referred with HSIL (cytology or biopsy, according to the two-tiered The Lower Anogenital Squamous Terminology)^17^ to the Women's Hospital of the University of Campinas (CAISM/Unicamp), when the conservative management was offered and agreed, from 2012 to 2019. It is a hospital for breast and gynecology cancer treatment in the center of São Paulo State, Brazil, referral for a population of ∼1.5 million women. All care in the service is free of charge, financed by the Brazilian public health system, the University, and research agencies. HPV vaccine in Brazil started in 2014 in 12-year-old girls, so in this cohort, women were not vaccinated.

The CAISM follows the Brazilian national guidelines that recommend conservative management for 24 months in patients with HSIL under 25 years, with follow-up by cytology and colposcopy at intervals of 4–6 months until two consecutive negative tests (no abnormal findings in colposcopy and cytology). If the lesion persists over 24 months or if the lesion progresses, or if the patient requests, excisional treatment were performed.^4^ In CAISM, conservative management is also offered to women aged 25–30 years when follow-up is warranted. This decision is shared with the patient after informed consent.

Women were referred by primary health care (PHC) or gynecology clinics from surrounding cities or Campinas. During the main study period, PHC smears from the region were executed by the cytology laboratory of CAISM, which returned the results to the city of origin for proper management. It is very likely that almost all samples for cytology collected in PHC were analyzed by the cytology laboratory of CAISM. The Ministry of Health qualifies the laboratory as one of the senior laboratories to perform quality control audits in Sao Paulo State. Routinely 30% of negative results were randomly selected and submitted for rapid review, and 10% for a full review by senior cytotechnologists. A pathologist reviews abnormal results. Local laboratories executed biopsies from PHC and gynecology clinics. The university pathology laboratory executed biopsies from CAISM. At the university pathology laboratory, all HSIL in women younger than 25 years and doubtful cases were confirmed by p16 immunohistochemical analysis.

Data were collected through medical records review. All women aged 30 or younger referred to CAISM by cytology or biopsy with an HSIL result were accessed (*n* = 335 records). Of these records, 204 were those of women whose management was chosen as active (excisional treatment) at admission/first or second visit. Patients with immunological deficiencies (AIDS, lupus erythematosus, or others) were excluded (*n* = 37 women). Pregnant women were excluded if they were pregnant from the diagnosis to the outcome but not if the pregnancy occurred during the follow-up time after the outcome.

Five patients whose treatment was chosen as “conservative” were considered lost to follow-up (less than two follow-ups after the initial diagnosis): one did not show up after the admission (first visit), when a biopsy was performed and confirmed HSIL; the other four patients did not show up after the first follow-up (second visit)—in one a biopsy was performed and showed LSIL; the second showed a cytology LSIL; the third showed a cytology negative; in the fourth, there was only the registry of a negative/normal colposcopy.

The diagnosis of HSIL was defined when the patient presented this result in cytology or biopsy. During admission and in every follow-up, conventional cytology sample was collected, and colposcopy performed. Biopsies were taken when colposcopy was suggestive of HSIL. The current practice is that in every “biopsy procedure” more than one fragment was taken, guided by the colposcopy.^18^ During persistent time, the excisional procedure could be performed by patient's request, after informed consent about procedure's risks and benefits. In these cases, the patient was censored. No progression to microinvasive or invasive cancer was observed in this sample.

Outcome definitions are given in Table 1. Other variables evaluated were age at diagnosis, parity, age of first sexual intercourse, hormonal contraceptive use and smoking habitat checked at admission, colposcopy impression at admission, time to outcome, and time of follow-up.

**Table 1.** Outcomes Definitions Used in the Study

Outcome	Definition
Regression	No evidence of HSIL in cytology or biopsy in at least two consecutive tests. The date of the regression for ‘time to outcome’ and survival analysis was the date when the second test was performed
Persistence	Persistence of HSIL in cytology or biopsy. The date of the persistence for ‘time to outcome’ was the date of the last follow-up or the date when an excisional procedure was indicated
Relapse	When cytology or biopsy showed a new HSIL after the date of regression.

HSIL, high-grade squamous intraepithelial lesions.

For comparisons of variables, chi-square or Fisher's exact test and Mann–Whitney test were used owing to the absence of normal distribution. Kaplan–Meyer method was used to calculate the regression probability. COX regression analysis was performed to identify factors related to regression, using stepwise criteria for multivariate analysis. The level of significance adopted for the statistical tests was 5% (*p* < 0.05). The “Ethics Committee of the University of Campinas” approved the study (CAAE: 93602418.4.0000.5404), and a consent form was provided to those women under follow-up during the research period.

## Results

**FIG. 1.**
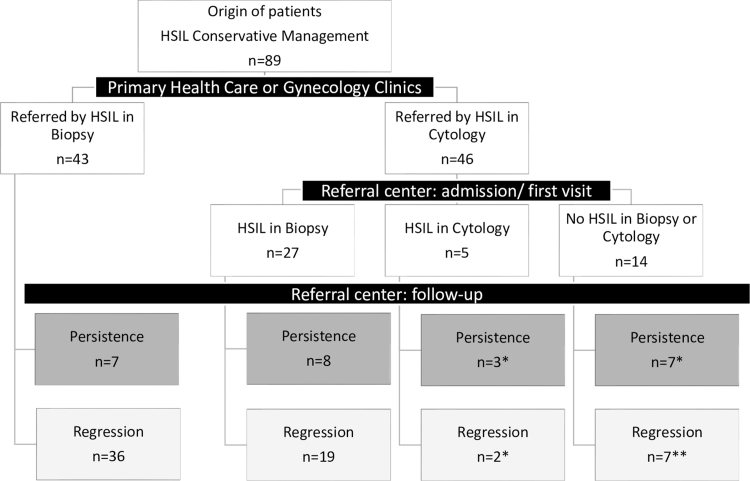
Flowchart of HSIL diagnosis or suspicious in 89 women under 30 years old managed conservatively. *At least one HSIL biopsy result during follow-up; **three of those women did not present an HSIL biopsy result during follow-up. HSIL, high-grade squamous intraepithelial lesions.

During the study period, 94 patients with HSIL at admission were managed conservatively, and five were lost to follow-up. Of the 89 patients included, no progression to microinvasive or invasive cancer was observed in this sample. Sixty-one (69%) were younger than 25 years, and 28 were aged 25–30 years (31%). A flowchart showing how HSIL was diagnosed or suspected (biopsy or cytology) is given in Figure 1.

The differences between groups were not significant in any of the analyzed variables (Table 2). Spontaneous regression was seen in 64 (72%) and persistence in 25 (28%). The average time to the outcome was 15.4 months (standard deviation [SD] = 7.7) in the regression group and 16.1 months (SD = 10.4) in the persistence group (*p* = 0.963). The follow-up time was 31.6 months (SD = 19.0) in the regression group and 27.2 months (SD = 22.8) in the persistence group (*p* = 0.885). The COX regression analysis showed no significant variable influencing regression on univariate and multivariate analysis (data not shown).

**Table 2.** Characteristics of 89 Women Under 30 Years Old Regarding Regression or Persistence of High-Grade Squamous Intraepithelial Lesions After Conservative Management

	Outcome		Total	*p*-value
	Regression	Persistence		
	Mean (SD)	Mean (SD)	Mean (SD)	
Age	22.6 (3.7)	22.2 (4.3)	22.5 (3.8)	0.760
Time to outcome	15.4 (7.7)	16.1 (10.4)	15.6 (8.4)	0.933
Follow-up time	31.6 (19.0)	27.2 (22.8)	31.5 (18.9)	0.885

	*n* (%)	*n* (%)	*N*	*p*-value
Age group				
<25 years	46 (73.7)	15 (26.3)	61 (100.0)	0.690
25–30 years	20 (71.4)	8 (28.6)	28 (100.0)	
Parity				
Nullipara	42 (72.4)	16 (27.6)	68 (100.0)	0.607
+1 birth	24 (77.4)	7 (22.6)	31 (100.0)	
First sexual intercourse^a^				
<15 years	17 (73.9)	6 (26.1)	23 (100.0)	0.934
≥15 years	46 (73.0)	17 (27.0)	65 (100.0)	
Smoking^a^				
Yes	8 (61.5)	5 (38.5)	13 (100.0)	0.312
No	57 (76.0)	18 (24.0)	75 (100.0)	
Hormonal contraception^a^				
Yes	42 (75.0)	14 (25.0)	56 (100.0)	0.748
No	23 (71.9)	9 (28.1)	32 (100.0)	
Colposcopy impression^a^				
Negative	15 (75.0)	5 (25.0)	20 (100.0)	0.963
Low grade	37 (75.5)	12 (24.5)	49 (100.0)	
High grade	13 (72.2)	5 (27.8)	18 (100.0)	

^a^ Missing information.

*p*-value, Chi-square or Fisher test, and Mann–Whitney test; SD, standard deviation.

**FIG. 2.**
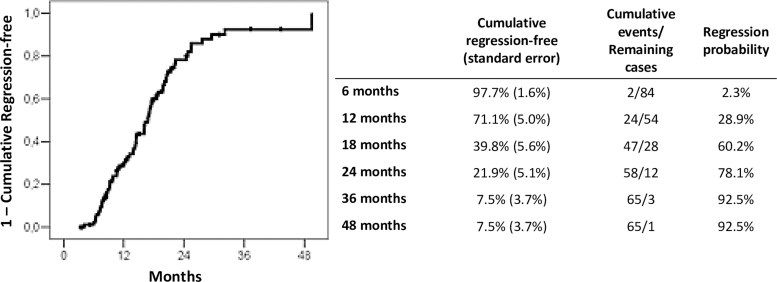
Kaplan–Meyer regression curve of HSIL conservative management in 89 women under 30 years old. The figure provides the regression curve of women with a HSIL result that were managed conservatively. The regression probability in 12, 18, and 24 months were, respectively, 28.9%, 60.2%, and 78.1%.

In Figure 2 the Kaplan–Meyer curve of the 89 patients to the event “Regression” is given. The curve shows that after 12, 18, and 24 months of conservative management, the regression rates were, respectively, 28.9%, 60.2%, and 78.1%. The figure indicates that most of the events happened between 12 and 18 months of follow-up.

Some clinical aspects of the regression are given in Table 3. We evaluate a subgroup of patients whose outcome “regression” had happened up to 12 or 24 months. Most of the patients had a biopsy confirming the initial diagnosis. In addition, most patients had the final cytology or biopsy result as negative for neoplasia after the outcome. In this analysis, a relapse (Table 1 definition) was observed in seven patients (12.3%). No differences were observed in having the outcome earlier or later regarding those variables. The average number of biopsy procedures performed until the outcome was 1.14 in the 12 months group and 1.12 in the 24 months group (*p* = 0.946).

**Table 3.** Clinical Aspects of Regression in Conservative Management of High-Grade Squamous Intraepithelial Lesions in 57 Women Under 30 Years Old

	Time to regression		*p*-value
	12 months	24 months	
	*n* (%)	*n* (%)	
Total	23 (100.0)	34 (100.0)	
Biopsy at diagnosis			
Yes	17 (73.9)	19 (55.9)	0.166
No	06 (26.1)	15 (44.1)	
Outcome			
Negative	16 (69.6)	24 (70.6)	0.934
ASC-US/LSIL	07 (30.4)	10 (29.4)	
Relapse (new HSIL)			
Yes	01 (04.3)	06 (17.7)	0.223
No	22 (95.7)	28 (82.3)	

	Mean (SD)	Mean (SD)	*p*-value
Biopsy procedures^a^ to outcome	1.14 (0.56)	1.12 (0.64)	0.946

^a^ Usually, more than one fragment is undertaken in every procedure.

ASC-US, atypical squamous cells of undetermined significance; LSIL, low-grade squamous intraepithelial lesions; *p*-value, chi-square or Fisher test.

## Discussion

This study analyzed 89 patients up to 30 years old with HSIL diagnosis managed conservatively and spontaneous regression was observed in 63 patients (70.8%). Most of the events happened between 12 and 18 months of follow-up. After 24 months, the regression rate was 78.1%. No clinical variable was relevant to influencing regression. Relapse was observed in seven patients (12.3%).

The high rate of regression observed, of 70.8%, is in line with the evidence in the literature.^11,12,19,20^ Regression rates were similar in the two groups evaluated: 73.7% in those younger than 25 years and 71.4% in those aged 25–30 years (*p* = 0.690). HPV infection in young women has a transitory nature.^8,15,21^ Most cases of HSIL diagnosed in this group correspond to CIN 2, which presents a behavior more similar to that of low-grade lesions.^10,15^ These cases may correspond to subtypes with lower oncogenic potential, such as others than 16 and 18.^22^ In this study, genotyping was not performed.

Clearance of HPV usually occurs from 12 to 24 months after infection, with cytological regression occurring at an average of 6 months before clearance.^1,2^ In this study, regression probabilities were 28.9% at 12 months, 60.2% at 18 months, and 78.1% at 24 months. The average time to the outcome was 15.4 months. The recommendation to follow these women for up to 24 months seems appropriate. Those who do not show regression after this period may correspond to the more oncogenic types of HPV or an immune deficiency.

After at least two consecutive negative tests, 7 of 57 (12.3%) in 24 months were found to have a recurrence. Studies on HSIL recurrence mainly refer to patients actively managed with excisional treatment and point to age, HPV type, smoking, and immunocompromised as the most relevant factors related to relapse. This risk persists for at least 25 years.^22(p16),23,24^ The HPV subtype has a significant influence on its natural history. HPV 16 is more associated with lesion persistence than other subtypes.^22^ The HPV typing can help decide the conservative management in those where the evidence is still controversial, like those from 25 to 30 years old and those immunocompromised.

The number of biopsy procedures in the follow-up of these patients did not influence the time for regression. The classic study by McCredie et al., which compared the long-term risk of invasive cervical cancer in women with CIN 3 who underwent serial biopsies with those who had initial conservative treatment, demonstrated a higher risk of disease persistence/development of invasive neoplasia in the later group.^3^ We did not find it, perhaps because of the reduced number of patients. However, this difference can be explained by the age discrepancy between the groups of the two studies: whereas the first study analyzed women with a mean age of 38 years, our sample analyzed only women under the age of 30 years. Our data suggest that follow-up biopsy can be reserved only for suspected progression in this group.

Age is recognized as a relevant factor influencing regression. Zhang and Lu, performing a meta-analysis of 16 articles, reported that age was inversely associated with the regression outcome.^25^ In this work, by including only young patients, we sought to identify some other factors that influenced the time and rate of regression. In the COX analysis, no clinical variable was significant influencing the outcome risk. In the meta-analysis by Bekos et al., the variables smoking, contraception, number of sexual partners, and socioeconomic status were relevant.^16^ Brun et al. found no relation in age, smoking, and regression.^26^ In this study, the inclusion of young patients may have been a selection bias to some variables such as parity and smoking, as their prevalence is low in this group.

This is a retrospective study, so interventions could not be fully controlled. Its main advantage is having included a reasonable number of patients in the same service, enabling uniform data collection. Furthermore, as a university service, the quality controls and care protocols are pretty accurate.

The main limitation is that in 19 cases there was no confirmatory biopsy of the HSIL cytological result at admission. However, only three of those women did not present at least one HSIL biopsy during the follow-up period (Fig. 1). The short follow-up period can also be interpreted as a limitation. HPV testing in Brazil is available only in some private clinics or for specific research projects.^27^ The women in our sample did not have access to this technology. Considering that HPV testing in women under 30 years is controversial because of its low sensitivity in this group, it is unlikely that the recommendations will be changed in the next years.^1,28^ Future researchers will define the role of genotyping in progression risk in this group of women.

## Conclusion

Spontaneous regression was observed in 70% of women younger than 30 years with HSIL managed conservatively. Most of the events happened between 12 and 18 months of follow-up. No clinical variable was relevant to influencing regression. Conservative management in this group is safe and should be the preferable approach.

## Data Availability

The datasets generated during this study are available from the corresponding author on reasonable request. Data records are submitted to specific restrictions to preserve confidentiality, although aggregate data may be shared upon request.

## Abbreviations Used

ASC-US: atypical squamous cells of undetermined significance
CIN: cervical intraepithelial neoplasia
HPV: human papillomavirus
HSIL: high-grade squamous intraepithelial lesions
LLETZ: large loop excision of the transformation zone
LSIL: low-grade squamous intraepithelial lesions
PHC: primary health care
SD: standard deviation

## References

[1] Schiffman M, Doorbar J, Wentzensen N, et al. Carcinogenic human papillomavirus infection. Nat Rev Dis Primers 2016;2:16086. 27905473 10.1038/nrdp.2016.86

[2] de Sanjosé S, Brotons M, Pavón MA. The natural history of human papillomavirus infection. Best Pract Res Clin Obstet Gynaecol 2018;47:2–13. 28964706 10.1016/j.bpobgyn.2017.08.015

[3] McCredie MRE, Sharples KJ, Paul C, et al. Natural history of cervical neoplasia and risk of invasive cancer in women with cervical intraepithelial neoplasia 3: A retrospective cohort study. Lancet Oncol 2008;9:425–434. 18407790 10.1016/S1470-2045(08)70103-7

[4] Instituto Nacional de Câncer. Diretrizes Brasileiras para o Rastreamento do Câncer do Colo do Útero 2016—segunda edição, 2016. Available at: https://www.inca.gov.br/sites/ufu.sti.inca.local/files/media/document/diretrizesparaorastreamentodocancerdocolodoutero_2016_corrigido.pdf Accessed June 3, 2022.

[5] Perkins RB, Guido RS, Castle PE, et al. 2019 ASCCP risk-based management consensus guidelines for abnormal cervical cancer screening tests and cancer precursors. J Low Genit Tract Dis 2020;24:102–131. 32243307 10.1097/LGT.0000000000000525 PMC7147428

[6] Kyrgiou M, Arbyn M, Bergeron C, et al. Cervical screening: ESGO-EFC position paper of the European Society of Gynaecologic Oncology (ESGO) and the European Federation of Colposcopy (EFC). Br J Cancer 2020;123:510–517. 32507855 10.1038/s41416-020-0920-9 PMC7434873

[7] Kyrgiou M, Athanasiou A, Kalliala IEJ, et al. Obstetric outcomes after conservative treatment for cervical intraepithelial lesions and early invasive disease. Cochrane Database Syst Rev 2017;11:CD012847. 29095502 10.1002/14651858.CD012847 PMC6486192

[8] Piris S, Bravo V, Alvarez C, et al. Natural history of histologically moderate cervical dysplasia in adolescent and young women. Onco Targets Ther 2014;7:2101–2106. 25419148 10.2147/OTT.S69776 PMC4235504

[9] de Sanjose S, Quint WG, Alemany L, et al. Human papillomavirus genotype attribution in invasive cervical cancer: A retrospective cross-sectional worldwide study. Lancet Oncol 2010;11:1048–1056. 20952254 10.1016/S1470-2045(10)70230-8

[10] Moscicki AB, Cox JT. Practice improvement in cervical screening and management (PICSM): Symposium on management of cervical abnormalities in adolescents and young women. J Low Genit Tract Dis 2010;14:73–80. 20043357 10.1097/lgt.0b013e3181cec411 PMC3058950

[11] Skorstengaard M, Lynge E, Suhr J, Napolitano G. Conservative management of women with cervical intraepithelial neoplasia grade 2 in Denmark: A cohort study. BJOG 2020;127:729–736. 31880054 10.1111/1471-0528.16081 PMC7383715

[12] Dempster-Rivett K, Innes CR, Simcock BJ, et al. Evaluation of guidelines for observational management of cervical intraepithelial neoplasia 2 in young women. Am J Obstet Gynecol 2020;223:408.e1–408.e11. 32109465 10.1016/j.ajog.2020.02.029

[13] Chevreau J, Mercuzot A, Foulon A, et al. Impact of age at conization on obstetrical outcome: A case-control study. J Low Genit Tract Dis 2017;21:97–101. 28157826 10.1097/LGT.0000000000000293 PMC5367499

[14] Australian Institute of Health and Welfare. National Cervical Screening Program: Guidelines for the management of screen-detected abnormalities, screening in specific populations and investigation of abnormal vaginal bleeding. Australian Institute of Health and Welfare. Available at: https://wiki.cancer.org.au/australia/Guidelines:Cervical_cancer/Screening Accessed March 24, 2021.

[15] Vale DB, Westin MC, Zeferino LC. High-grade squamous intraepithelial lesion in women aged <30 years has a prevalence pattern resembling low-grade squamous intraepithelial lesion. Cancer Cytopathol 2013;121:576–581. 23765869 10.1002/cncy.21312

[16] Bekos C, Schwameis R, Heinze G, et al. Influence of age on histologic outcome of cervical intraepithelial neoplasia during observational management: Results from large cohort, systematic review, meta-analysis. Sci Rep 2018;8:6383. 29686397 10.1038/s41598-018-24882-2 PMC5913272

[17] Darragh TM, Colgan TJ, Thomas Cox J, et al. The Lower Anogenital Squamous Terminology Standardization project for HPV-associated lesions: Background and consensus recommendations from the College of American Pathologists and the American Society for Colposcopy and Cervical Pathology. Int J Gynecol Pathol 2013;32:76–115. 23202792 10.1097/PGP.0b013e31826916c7

[18] Associação Brasileira de Patologia do Trato Genital Inferior e Colposcopia. Laudo Colposcópico. Available at: https://colposcopia.org.br/ Accessed June 3, 2022.

[19] Díaz Del Arco C, Jiménez Ayala B, García D, Sanabria C, Fernández Aceñero MJ. Distribution of cervical lesions in young and older women. Diagn Cytopathol 2019;47:659–664. 31184808 10.1002/dc.24163

[20] Discacciati MG, de Souza CAS, d'Otavianno MG, et al. Outcome of expectant management of cervical intraepithelial neoplasia grade 2 in women followed for 12 months. Eur J Obstet Gynecol Reprod Biol 2011;155:204–208. 21193261 10.1016/j.ejogrb.2010.12.002

[21] Bano F, Kolhe S, Zamblera D, et al. Cervical screening in under 25s: A high-risk young population. Eur J Obstet Gynecol Reprod Biol 2008;139:86–89. 18029085 10.1016/j.ejogrb.2007.08.020

[22] Loffredo D'Ottaviano MG, Discacciati MG, Andreoli MA, et al. HPV 16 is related to the progression of cervical intraepithelial neoplasia grade 2: A case series. Obstet Gynecol Int 2013;2013:328909. 24369469 10.1155/2013/328909 PMC3867922

[23] Fernández-Montolí ME, Tous S, Medina G, Castellarnau M, García-Tejedor A, de Sanjosé S. Long-term predictors of residual or recurrent cervical intraepithelial neoplasia 2-3 after treatment with a large loop excision of the transformation zone: A retrospective study. BJOG 2020;127:377–387. 31631477 10.1111/1471-0528.15996

[24] Egemen D, Cheung LC, Chen X, et al. Risk estimates supporting the 2019 ASCCP risk-based management consensus guidelines. J Low Genit Tract Dis 2020;24:132–143. 32243308 10.1097/LGT.0000000000000529 PMC7147417

[25] Zhang J, Lu CX. Spontaneous regression of cervical intraepithelial neoplasia 2: A meta-analysis. Gynecol Obstet Invest 2019;84:562–567. 31055567 10.1159/000497286

[26] Brun JL, Letoffet D, Marty M, Griffier R, Ah-Kit X, Garrigue I. Factors predicting the spontaneous regression of cervical high-grade squamous intraepithelial lesions (HSIL/CIN2). Arch Gynecol Obstet 2021;303:1065–1073. 33175197 10.1007/s00404-020-05853-3

[27] Carvalho CF, Teixeira JC, Bragança JF, Derchain S, Zeferino LC, Vale DB. Cervical cancer screening with HPV testing: Updates on the recommendation. Rev Bras Ginecol Obstet 2022;44:264–271. 35170010 10.1055/s-0041-1739314 PMC9948069

[28] Koliopoulos G, Nyaga VN, Santesso N, et al. Cytology versus HPV testing for cervical cancer screening in the general population. Cochrane Database Syst Rev 2017;8:CD008587. 28796882 10.1002/14651858.CD008587.pub2 PMC6483676

